# An Induced Pocket for the Binding of Potent Fusion Inhibitor CL-385319 with H5N1 Influenza Virus Hemagglutinin

**DOI:** 10.1371/journal.pone.0041956

**Published:** 2012-08-02

**Authors:** Runming Li, Deshou Song, Zhibo Zhu, Hanhong Xu, Shuwen Liu

**Affiliations:** 1 School of Pharmaceutical Sciences, Southern Medical University, Guangzhou, China; 2 State Key Laboratory for Conservation and Utilization of Subtropical Agro-Bioresources, Key Laboratory of Natural Pesticides and Chemical Biology, South China Agricultural University, Guangzhou, China; UMR-S665, INSERM, Université Paris Diderot, INTS, France

## Abstract

The influenza glycoprotein hemagglutinin (HA) plays crucial roles in the early stage of virus infection, including receptor binding and membrane fusion. Therefore, HA is a potential target for developing anti-influenza drugs. Recently, we characterized a novel inhibitor of highly pathogenic H5N1 influenza virus, CL-385319, which specifically inhibits HA-mediated viral entry. Studies presented here identified the critical binding residues for CL-385319, which clustered in the stem region of the HA trimer by site-directed mutagenesis. Extensive computational simulations, including molecular docking, molecular dynamics simulations, molecular mechanics generalized Born surface area (MM_GBSA) calculations, charge density and Laplacian calculations, have been carried out to uncover the detailed molecular mechanism that underlies the binding of CL-385319 to H5N1 influenza virus HA. It was found that the recognition and binding of CL-385319 to HA proceeds by a process of “induced fit” whereby the binding pocket is formed during their interaction. Occupation of this pocket by CL-385319 stabilizes the neutral pH structure of hemagglutinin, thus inhibiting the conformational rearrangements required for membrane fusion. This “induced fit” pocket may be a target for structure-based design of more potent influenza fusion inhibitors.

## Introduction

Enveloped viruses transfer their genetic material into cells by fusing their viral membrane with cellular membranes. Membrane fusion is catalyzed by virus-specific envelope proteins. In general, these viral envelope proteins have a common requirement for structural rearrangements during the fusion process. These conformational changes can be triggered by different stimuli, including acidification in low pH environment, as well as receptor and non-receptor binding. Upon activation, envelope proteins insert a hydrophobic “fusion peptide” into the target membrane to bridge the gap between viral and cellular membranes, promoting the process of membrane fusion [1]–[5]. Depending on the structural characteristics, viral fusion proteins can be categorized into three classes. Class I fusion proteins are characterized by trimeric helical bundles, such as influenza A hemagglutinin or HIV gp120/gp41. Class II fusion proteins have an abundance of ß-sheets, such as Dengue virus protein E and Semliki forest virus protein E1. Class III fusion proteins are characterized by a combination of helical bundles and ß-sheets, including vesicular stomatitis virus protein G (VSV-G), baculovirus fusion protein gp64, and others [5]. Influenza virus hemagglutinin is one of the best-characterized viral proteins which can mediate membrane fusion between influenza virus and target cell.

Hemagglutinin (HA) is initially expressed as a precursor, HA0, which is proteolytically cleaved into the functional HA1 and HA2 subunits, linked by a single disulfide bond. After receptor binding, the virus will be uptaken into cells by endocytosis. Within the endosome, the virion is exposed to the acid pH condition [2], [3], triggering the HA protein to undergo an irreversible conformation change from its metastable pre-fusion conformation to a low-pH hairpin structure. The resulting extrusion of the “fusion peptide” (FP) from the interior of the HA2 at the neutral-pH condition toward the endosomal membrane promotes the fusion of the viral and endosomal membranes [6], [7]. X-ray crystallographic studies have demonstrated the extensive rearrangement of residues in HA2 at low pH with respect to their relative orientation and coil-coil formation, loop-to-helix or helix-to-loop transition [1].

Mutagenesis of hemagglutinin has resulted in the identification of many key residues affecting the process of fusion. These residues are usually located in four regions, including the fusion peptide, the fusion peptide pocket, the coil-coil region of HA2 and the interface between HA1 and HA2 [6], [8]–[13]. However, these mutagenesis studies provided little information about the critical residues that initiate structural rearrangement required for membrane fusion at low pH environment. An important finding demonstrated that an arginine to histidine mutation of residue 106 in HA2 (R106_2_H mutation) could retain HA in a near pre-fusion conformation under fusogenic pH condition [14]. This finding indicates that the disruption of inter-subunit ionic interactions between HA1 membrane-distal domain and HA2 central helices is necessary to initiate the irreversible, fusion-related conformational changes [14].

The mechanism of membrane fusion mediated by hemagglutinin makes it a potential anti-influenza target. Several small molecules are able to block virus infection by inhibiting the conformational changes required for membrane fusion [15]–[17]. However, their binding sites on HA, the interaction between molecules and HA, as well as the contribution of such interaction to their antiviral activity, are still not clear. We previously found that CL-385319 could inhibit H5N1 influenza virus infection by blocking viral entry [18]. In the present work, we further identified the binding site of CL-385319 on HA by more detailed site-directed mutagenesis. Furthermore, computer-assisted programs were used to investigate the binding mechanism. Our result showed that CL-385319 could bind to a conserved interface in the stem region of HA. Since the interaction between CL-385319 and HA contribute to the stability of the HA, inhibitory activity against H5N1 influenza virus can be expected.

## Results

### Alanine Substitutions on Residues Surrounding M24_1_ and F110_2_ have no Influence on H5N1 Pseudovirus Generation

In a previous study, we found that either the M24_1_A mutation in HA1 or the F110_2_S mutation in HA2 results in resistance to CL-385319 [18]. These results strongly suggest that both M24_1_ and F110_2_ may be critical for CL-385319 binding. To further investigate potential key residues on HA involved in the interaction with CL-385319, we selected amino acids surrounding M24_1_ and F110_2_ located within a sphere of the radius of 3 Å, according to the available crystal structure of HA (pdb: 2IBX), including K43_2_, D46_2_, G47_2_,V48_2_, K51_2_, E105_2_, R106_2_ and T107_2_. These residues were substituted by alanine, and the analysis of these mutants was performed.

**Figure 1:**
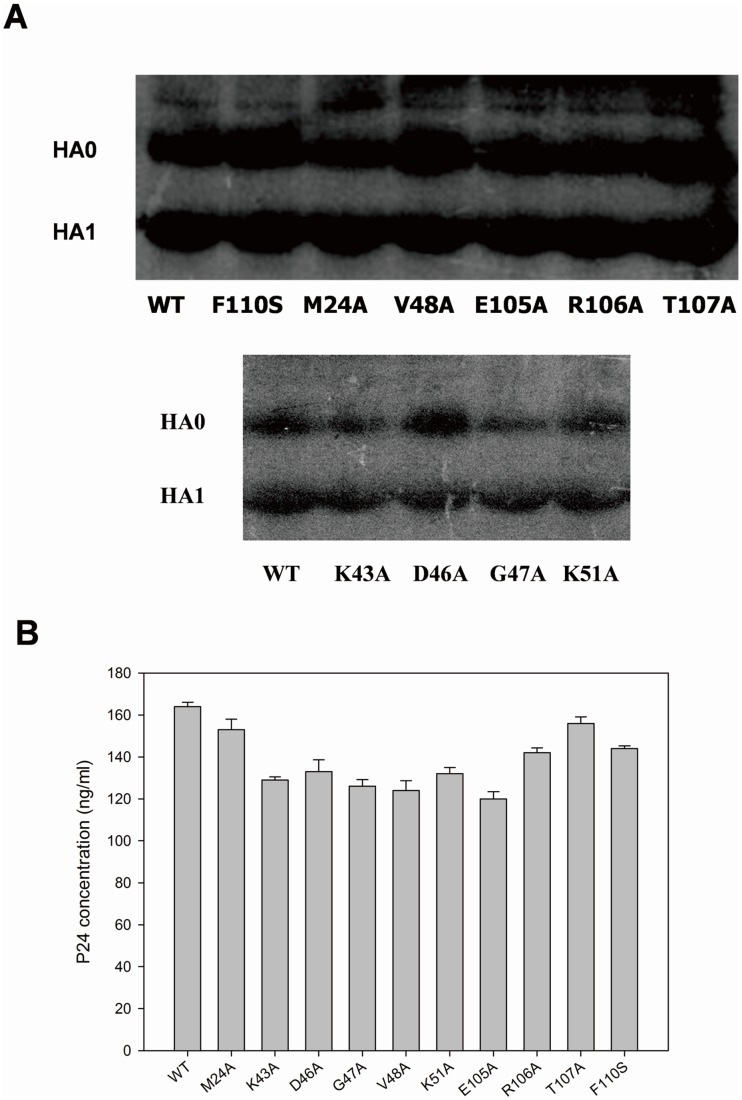
The expression of HA on the surface of wild-type and mutant pseudoviruses, as determined by Western blotting.

The expression of HA on the surface of the generated mutant pseudoviruses was studied by Western blotting. Wild-type and mutant pseudoviruses were lysed, and the HA expression was analyzed. As shown in Figure 1A, the expression of all HA proteins containing mutations were comparable to that of the wild type. The amount of p24 contained in the pseudovirus was also quantified using the HIV-1 p24 ELISA kit. The data showed that wild-type and mutant pseudoviruses contained a similar amount of p24 (Figure 1B). Taken together, these findings indicated that the alanine substitutions on residues surrounding M24_1_ and F110_2_ have no influence on H5N1 pseudovirus generation.

### Mutations of E105_2_ and T107_2_ on HA cause the Lost Infectivity of H5N1 Pseudovirus

**Figure 2:**
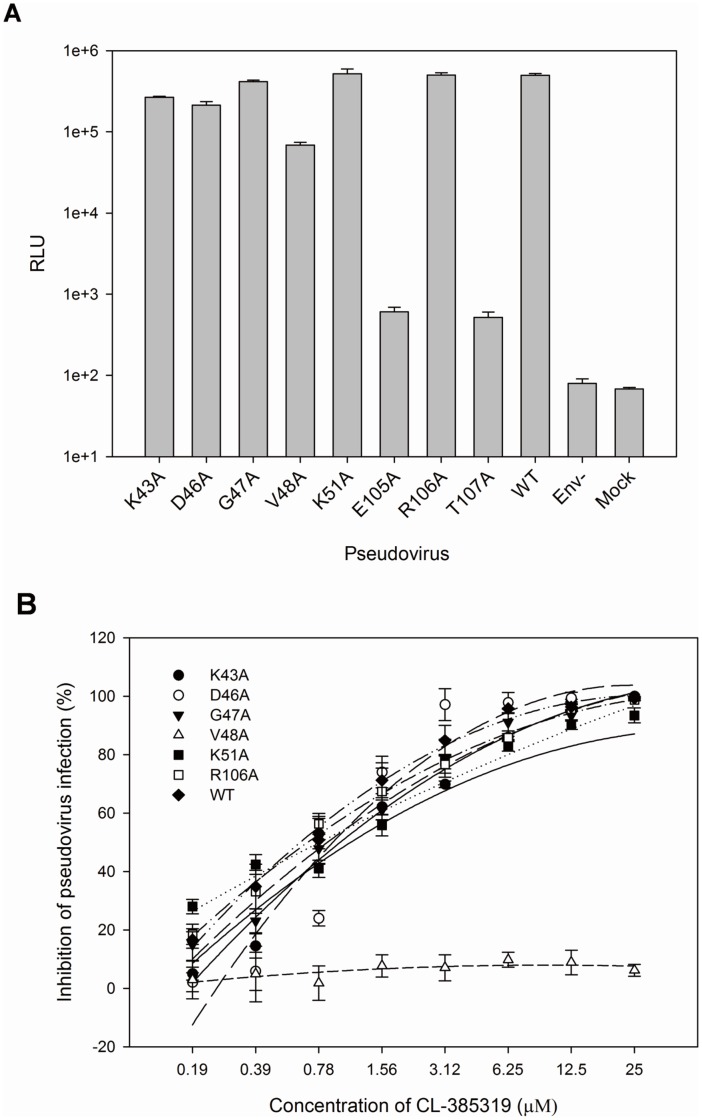
Site-directed mutagenesis analysis. A) The infectivities of mutant H5N1 pseudoviruses in MDCK cells. Wild-type pseudovirus was used as the positive control, while Env^-^ pseudovirus and cells-only (mock) were used as the negative control. B) Inhibitory activity of CL-385319 against the infection of the mutant H5N1 pseudoviruses. The samples were tested in triplicate, and the data were presented in mean ± SD. This experiment was repeated twice with similar results.

The effects of mutations on H5N1 pseudovirus infectivity were analyzed by single-cycle infection assay. MDCK cells were infected with mutant pseudoviruses, and the luciferase activity was detected 72 h post-infection. The E105_2_A and T107_2_A mutations eliminated the infection of pseudovirus, while the other mutations had no significant effects on pseudovirus infectivity (Figure 2A). These results indicated that the E105 and T107 on HA2 might be the key residues for H5N1 influenza virus infection.

### V48_2_, as well as M24_1_ and F110_2_, are Critical for the Interaction of CL-385319 with HA

In our previous study, we found that M24_1_ and F110_2_ are involved in the interaction with CL-385319 on H5-typed HA [18]. The pseudovirus containing M24_1_A or F110_2_S mutation was highly resistant to CL-385319. The IC_50S_ for inhibition of pseudovirus infection by CL-385319 were 1.50±0.13 µM, 106.31±6.71 µM and >100 µM for the wild-type, F110_2_S and M24_1_A-substituted pseudovirus, respectively. In the present study, we further found that the V48_2_A mutation also resulted in resistance to CL385319, with an IC_50_>100 µM. The remaining mutations, except E105_2_A and T107_2_A, had little effect on the antiviral activity of CL-385319 (Figure 2B). This result suggested that F110_2_, M24_1_, V48_2_ are critical for CL-385319 binding.

### Dynamic Stability from Molecular Docking

**Figure 3:**
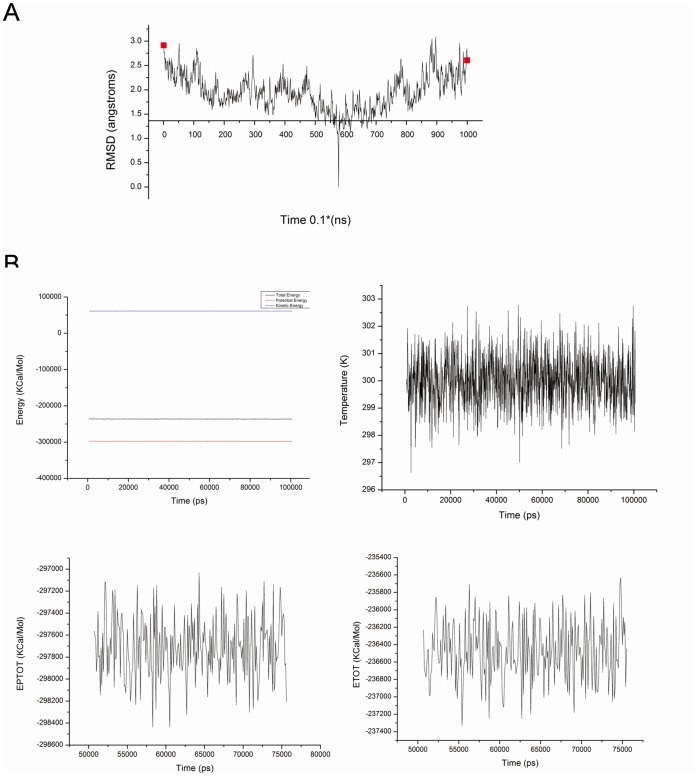
Dynamic stability from molecular docking simulation. A) The root-mean square deviation (RMSD) values with respect to the initial structures. B) The total energy (ETOT), potential energy (EPTOT),temperature (TEMP) and kinetic energy (EKTOT) fluctuations of the complex with the ligand binding versus simulation time.

To get a better understanding of the mechanism of CL-385319, we then conducted extensive computational simulations, first with molecular docking. Generally, it is important to ensure that molecular dynamics (MD) simulations are thoroughly equilibrated because obtaining a stable MD trajectory is essential for subsequent analysis. Therefore, the root-mean square deviation (RMSD) of the C^α^ atoms with reference to the initial structures over the 100-ns simulation times was determined (Figure 3A). Briefly, the RMSD of the protein backbone and inhibitor atoms stabilized around 1.50 Å from 50 ns to 75 ns, and the variation is within 1.0 Å. To further validate the equilibrium, we also analyzed the total energy (ETOT), potential energy (EPTOT), temperature (TEMP) and kinetic energy (EKTOT) fluctuations of the protein-inhibitor complex during the MD simulations (Figure 3B). All these data confirmed that the CL-385319-HA complex had achieved equilibrium from 50 ns to 75 ns after MD simulations.

### MM_GBSA Calculations Predict the Critical Residues

It is generally accepted that docking result needs to be further processed with more accurate methods. It should take into account the factors such as treatment of long-range electrostatics, desolvation of binding species and entropic contributions. With various levels of accuracy and computational expense, the computational approaches include free energy perturbation (FEP) [19], thermodynamic integration (TI) [20], linear response (LR) [21], molecular mechanics Poisson-Boltzmann surface area (MM_PBSA), and molecular mechanics generalized Born surface area (MM_GBSA) [22], [23]. In particular, MM_GBSA combine molecular mechanics and continuum solvent models to estimate ligand binding affinities and are faster by several orders of magnitude than FEP or TI. Many studies showed that FEP is indeed the most accurate method for the H5N1 antigen-receptor and antigen-antibody binding affinities [24]–[26]. Although not as accurate as FEP, it is proved that the correlations between MM-PBSA or MM-GBSA binding free energies and experimental affinities were excellent in most cases [27]. To explore the inhibition mechanisms of CL-385319 with respect to its interaction with HA at the atomic level, considering the laboratory computer resources and protein-ligand system (nearly 100,000 atoms), MM-GBSA method was carried out to calculate the binding free energies. The MM_GBSA calculation was constructed based a total of 250 snapshots that taken from the 50 ns to 75 ns MD production. Importantly, the calculated binding free energy of the complex was −36.9567 kcal mol^−1^, which indicated that the binding of CL-385319 to HA could increase the stability of HA protein.

Furthermore, the computational alanine scanning (CAS) method was applied to estimate the changes of the binding free energies resulting from the mutations on surrounding amino acid residues of the 50 ns to 75 ns MD production. The results are presented in Table 1. Residues showing a change of more than 1 kcal/mol in the binding free energy, when replaced by alanine, are defined as hot spots. As the predicted relative binding free energy of the alanine mutation increases, its contribution to CL-385319-HA complex stability correspondingly increases. From the CAS calculation, V48_2_, E103_2_, K51_2_, R106_2_, T107_2_, F110_2_ and M24_1_ were predicted as the critical residues for CL-385319 binding. The residues of V48_2_, F110_2_ and M24_1_ were also confirmed by our mutation assay against H5N1 psuedoviruse infection.

**Table 1:** Results of the CAS for HA main residues in CL-385319 binding (kcal/mol).

Residues	MM_GBSA		ΔΔ G
	Wild Type	Ala mutation	
V48_2_	−36.9567	−35.8552	1.1015
F110_2_	−36.9567	−31.9767	4.9800
M24_1_	−36.9567	−32.9503	4.0064
E105_2_	−36.9567	−36.8209	0.1358
R106_2_	−36.9567	−33.0456	3.9111
E103_2_	−36.9567	−35.8673	1.0894
T107_2_	−36.9567	−35.2272	1.7295
K51_2_	−36.9567	−35.4453	1.5114

### Charge Density and its Laplacian Calculations Predicted an “Induced Fit” Mechanism

**Figure 4:**
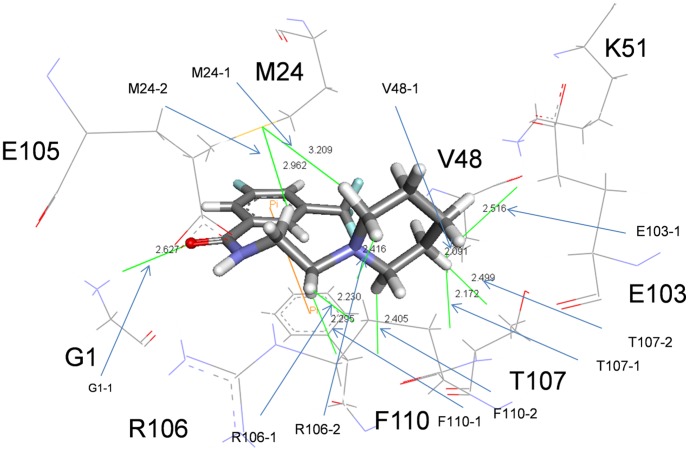
Hydrogen bonds formed between CL-385319 and residues in binding pocket.

**Figure 5:**
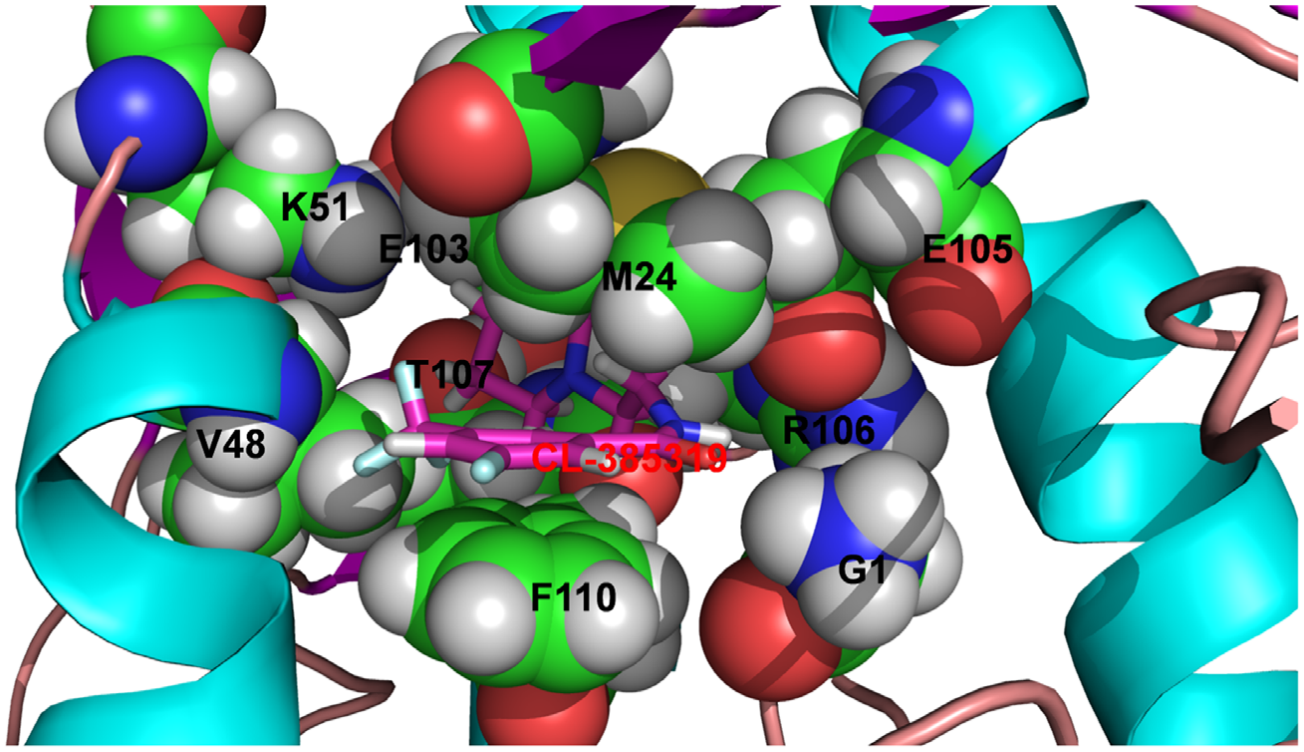
The lowest energy conformation of CL-385319-hemagglutinin complex.

**Figure 6:**
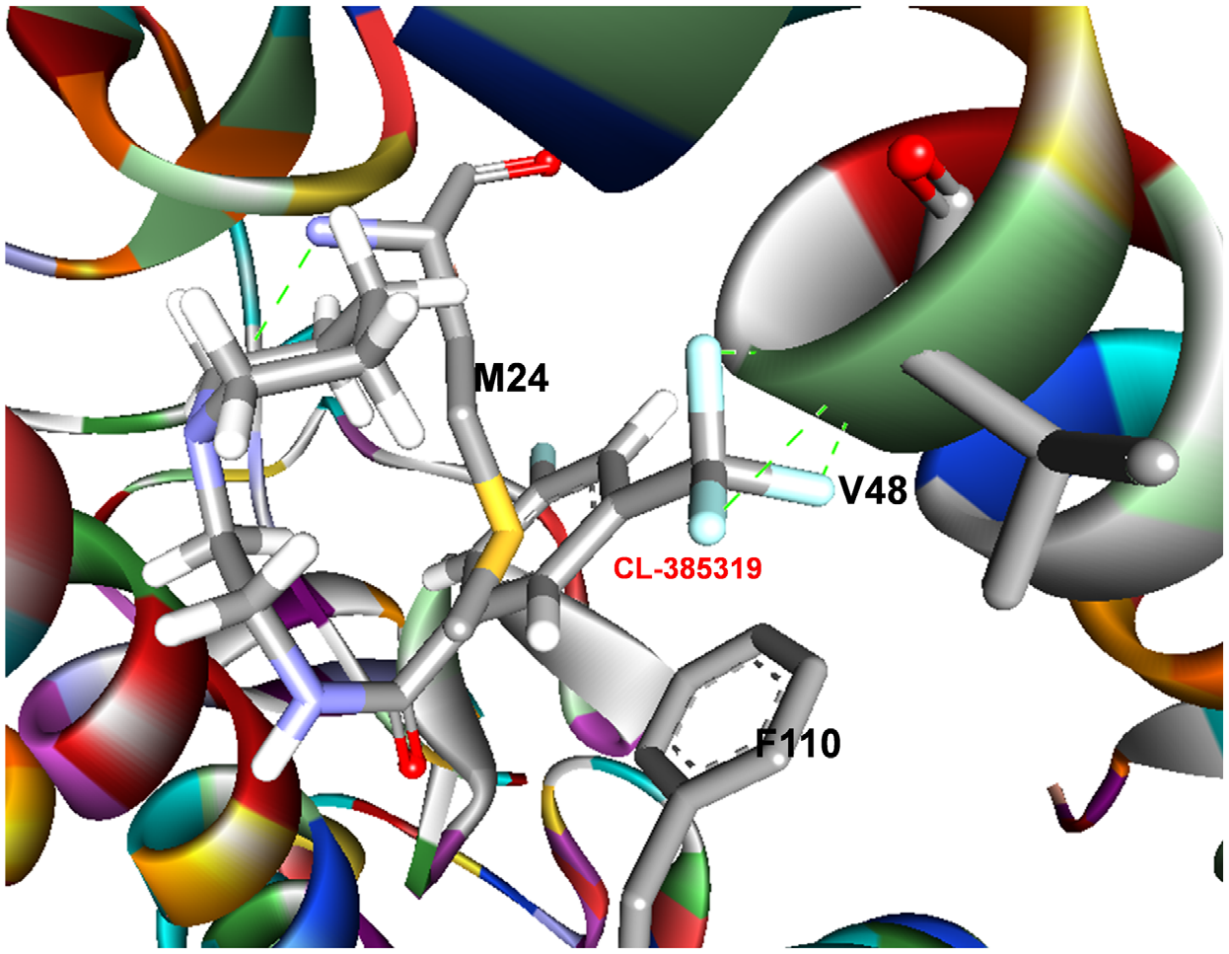
The initial structure of CL-385319-hemagglutinin complex.

The charge densities of the bond critical points of the lowest energy structure are listed in Table 2. All the charge densities are positive, which confirms their H-bonding property. M24_1_, G1_2_, R106_2_, F110_2_, E103_2_, V48_2_ and T107_2_ can form two, one, two, two, one, one and two hydrogen bonds with CL-385319, respectively (Figure 4). Arene-arene interactions play an essential role in the structure of biological macromolecules, as well as the interaction between ligand and protein [28]–[30]. As shown in Figure 5, the benzene ring of CL-385319 was sandwiched by F110_2_ and M24_1_ via π-π interactions. In the initial complex structure, the triﬂuorophenyl group of CL-385319 formed three H-bonds interaction with V48_2_, but these bonds were broken during MD simulation (Figure 6). We believe that this collapse of H-bonds resulted from the presence of π-π interactions mentioned above. Remarkably, the positions of side chain atoms of F110_2_, V48_2_ and CL-385319 remained the same as those of the initial structure during a 100-ns molecular dynamic simulation.

**Table 2:** Charge Density (**ρ**_b_) and Its Laplacian (**∇** ^2^ **ρ**_b_) at BCPs between Substrate and Main Residues at B3LYP/6-31+G (d,p) Level of Theory (a.u.).

Residues	Charge density (ρ_b_)	Laplacia (∇^2^ ρ_b_)
M24_1_-1	0.004592716	0.013105
M24_1_-2	0.008095317	0.027000
G1_2_-1	0.005061328	0.021324
R106_2_-1	0.006739404	0.022242
R106_2_-2	0.005960687	0.021456
F110_2_-1	0.005770899	0.021112
F110_2_-2	0.005861183	0.022037
T107_2_-1	0.003880408	0.013892
T107_2_-2	0.007995447	0.029168
E103_2_-1	0.004576331	0.016031
V48_2_-1	0.009776239	0.035476

**Figure 7:**
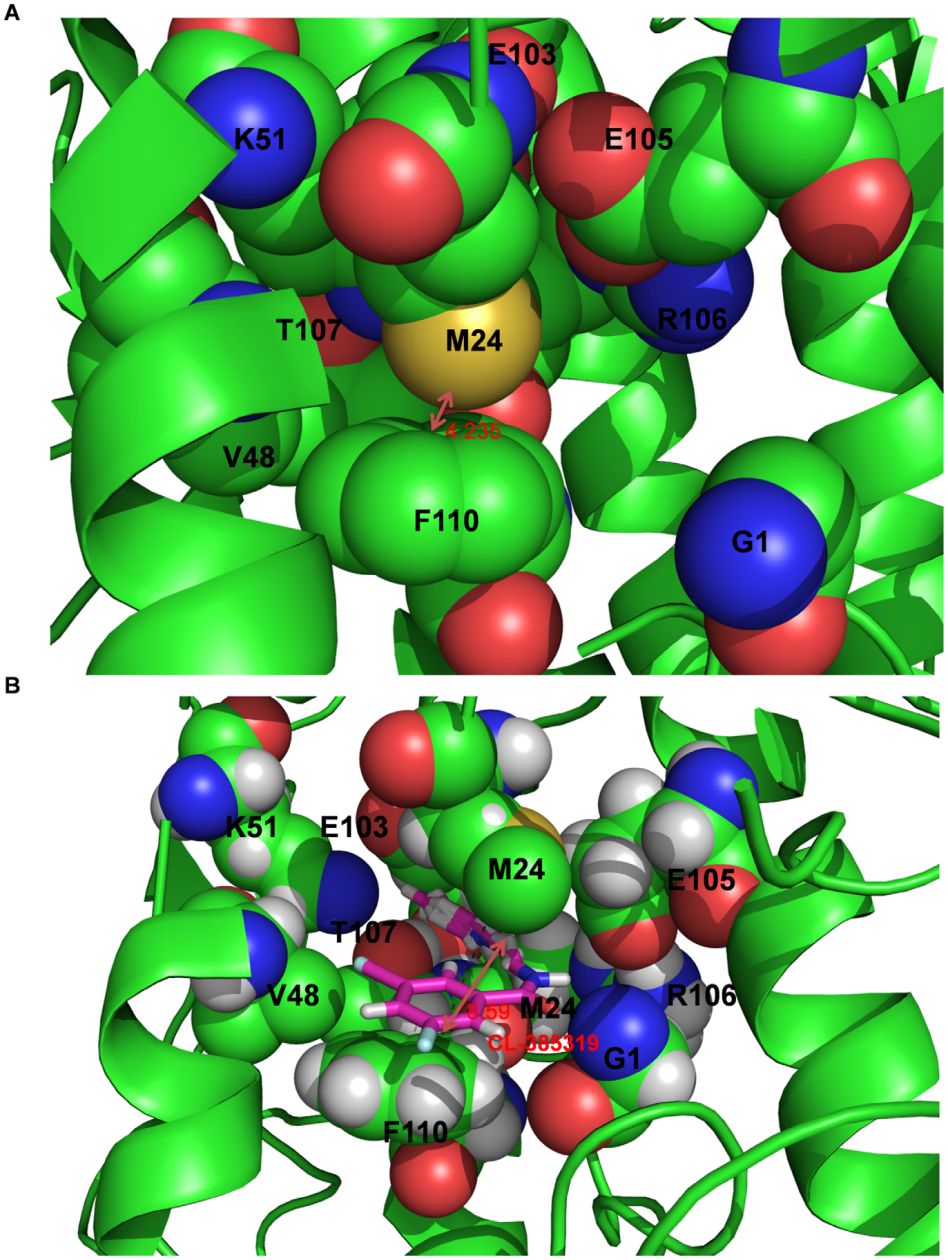
The averaged distance between residue F110_2_ and residue M24_1_. A) The initial conformation of CL-385319-hemagglutinin complex. B) The averaged conformation of CL-385319-hemagglutinin complex over MD simulation.

When we compared the averaged structure of 100 ns MD simulation with that of X-ray crystal structure (pdb: 2IBX), we found no evidence of an originally formed binding pocket, and the distance between F110_2_ and M24_1_ is 4.235 Å. However, comparison of the backbone and side-chain RMSD showed that the side-chain residue of M24_1_ significantly changed conformation by shifting away from F110_2_ to form the binding site. The averaged distance between F110_2_ and M24_1_ increased from 4.235 Å to 8.950 Å (Figure 7A and 7B), indicating that CL-385319 recognition and binding to HA is a process of “induced fit”. To detail the “induced fit” mechanism, a video composed of hundreds of snapshots through the whole MD simulation was produced (supplemental data, Movie S1). This video provides straight-forward information of the distance between F110_2_ and M24_1_ during MD simulation. At 1200 ps, the distance between F110_2_ and M24_1_ is 5.370 Å, a little larger than that observed from CL-385319-HA complex after energy minimization (5.330 Å), which means the benzene ring of CL-385319 can enter the pocket between F110_2_ and M24_1_. Along with the MD simulation, the distance between F110_2_ and M24_1_ had been increasing, as well as the distance between triﬂuorophenyl group of CL-385319 and V48_2_. Finally, the average distance between F110_2_ and M24_1_ residues increased to 8.950 Å. Other significant conformation changes of residues could not be observed during MD simulation.

## Discussion

Hemagglutinin (HA) mediates fusion of viral and endosomal membranes during the entry of influenza virus. After virus is uptaken into cells by endocytosis, endosomal acidification triggers HA2 refolding towards its hairpin-like post-fusion structure involving extrusion of the fusion peptide (FP) from the interior of the HA2 at the neutral-pH structure toward the endosomal membrane. Since this structural rearrangement is critical for viral entry, HA is a very promising anti-influenza drug target. Broad neutralizing antibodies that recognized the conserved region in the membrane-proximal stem of HA1 and HA2 could provide immunity to diverse influenza subtypes [1], [6]. Several small molecules, such as stachyflin [31], BMY-27709 [15], and CL-385319 [17], can prevent HA from undergoing low pH-induced conformational change. However, the molecular mechanism underlying the binding of these compounds to HA and the selection of specific amino acids from resistant virus are mostly still unclear.

In the present study, we identified the critical residues for CL-385319 binding on H5N1 hemagglutinin using site-directed mutagenesis analysis. We generated a series of mutant HA pseudoviruses and tested their susceptibility to the compound. Results showed that the M24_1_A, F110_2_S and V48_2_A mutations rendered the virus highly resistant to CL-385319, suggesting that M24_1_, F110_2_ and V48_2_ are the critical residues involved in the binding of CL-385319. M24_1_, F110_2_ and V48_2_ are highly conserved in all H5N1 strains. Interestingly, the individual E105_2_A and T107_2_A mutation eliminated pseudovirus infection, while Western blotting analysis showed that neither mutation affected HA protein expression on the surface of pseudovirus. These results suggest that E105_2_ and T107_2_ are critical for viral entry and that these mutations might interfere with the structural rearrangement needed for membrane fusion.

Furthermore, extensive computational simulations, including molecular docking, molecular dynamics (MD) simulations, MM_GBSA calculations, as well as charge density and its Laplacian calculations, have been carried out to uncover the detailed mechanism of CL-385319 binding to HA. The CL-385319 binding site is formed by HA1 and HA2 in one monomer. There are three identical binding sites per HA trimer. Consistent with the experimental mutagenesis result, the π−π interactions between CL-385319 and residues F110_2_ and M24_1_ make important contributions to the binding affinity. In addition, CL-385319 can form hydrogen bonds with the residues of E105_2_, R106_2_ and T107_2_.

As suggested by Koshland, the induced-fit model describes the capability of a proper ligand to induce specific structural change of the active site such that the protein is able to achieve the final key-lock state [32]. The “induced fit” model is generally accepted and widely applied to picture protein-protein interactions, especially favoring prolonged and strong interactions between ligand and protein [33]. Indeed, in the present study, we found that the binding of CL-385319 to HA is a process of “induced fit”. In the CL-385319-HA complex structure derived from molecular docking, which was used as starting structure for molecular dynamics simulation, the triﬂuorophenyl group of CL-385319 formed three important H-bonds interaction with V48_2_. The averaged structure of 100 ns MD simulation shows that the benzene ring of CL-385319 was sandwiched by F110_2_ and M24_1_ via π-π interactions, while the H-bonds interaction between triﬂuorophenyl group and V48_2_ was not observed in the lowest energy structure. Therefore, we believe that the H-bond interaction between the triﬂuorophenyl group and V48_2_ stabilizes the inhibitor in the early stage of MD and facilitates the formation of π-π interactions. Originally, no binding pocket was observed between F110_2_ and V48_2_. However, during MD simulation, a significant conformational change of the M24_1_ residue was observed, as it shifted away from F110_2_ to accommodate CL-385319. In addition, the free energy of CL-385319 binding to HA calculated by the MM_GBSA method showed that the binding process is thermodynamically favorable. Therefore, we conclude that CL-385319 binding to HA occurs through the induced-fit pathway.

The critical residues for CL-385319 binding locate in the stem region of HA. Ionic residues at or near this region are the only residues that experience different solvent environments before and after the insertion of fusion peptide into the cavity at the trimer interface, a necessary priming step of HA fusion. These residues are therefore suggested to play a critical role in fusion activation because of the apparent relationship between their chemical environment and HA sensitivity to low pH. Mutational analysis showed that amino acids in this cavity are associated with low pH-induced conformational change. For example, the E105_2_K mutation increased the stability of HA protein in low-pH [34], while the R106_2_H mutation improved the stability of H2-typed HA and inhibited the irreversible conformational rearrangement at fusogenic pH [14]. However, until now, these ionic residues had not been correlated with properties of influenza fusion inhibitors. Therefore, understanding how CL-385319 stabilizes HA could provide a clue for the development of new influenza fusion inhibitors. In H5N1-typed HA2, E103_2_, E105_2_, R106_2_ and T107_2_ are located in the stem region which experienced different environments as described above. Mutagenesis results showed that E105_2_A and T107_2_A mutation eliminated pseudovirus infectivity. Molecular dynamics simulation showed that CL-385319 has hydrogen-bonding interactions with residues of R106_2_ and T107_2_. These interactions might interfere with the disruptions of inter-subunit ionic interactions induced by low-pH. Meanwhile, CL-385319 has hydrogen-bonding interaction with residues of G1_2_, the first residue of HA fusion peptide. It is known that G1_2_E mutation abolish HA-mediated mutation [35]–[37]. CL385319 might stabilize the conformation of the fusion peptide through this interaction. In addition, the dissociation of HA1 from HA2 is the first step for HA conformational rearrangement. Previous study showed that CL-385319 can inhibit the proteolysis of purified H1-typed HA in low-pH [17]. We demonstrated that the π-π interaction between CL-385319 and the residue of F110_2_ and M24_1_ may provide a cross-linking of the HA trimer, thus inhibiting the dissociation of HA1 and HA2; even the HA0 was proteolyzed into HA1 and HA2. As a consequence, CL-385319 binding stabilizes the neutral pH conformation of HA, rendering its membrane fusion inactive.

In conclusion, we identified the critical residues required for CL-385319 binding to hemagglutinin by site-directed mutagenesis. Autodock combined with molecular dynamics simulation was used to elucidate the three-dimensional structure of CL-385319 bound to the active sites. MD simulation revealed an optimal conformation of CL-385319-HA complex, in which the inhibitor forms a sandwich π-π interaction with residues F110_2_ and M24_1_ and forms several H-bonds with residues in the binding pocket. MD simulation also revealed that the binding pocket, which does not exist originally, is formed by the interaction between CL-385319 and HA. That is to say, CL-385319 binding to HA occurs through the induced-fit pathway. Moreover, MD simulation showed that this binding mode could stabilize the neutral pH conformation of HA. We believe that this property is important for antiviral activity of CL-385319. The structural and mechanistic insights from the present study provide a valuable foundation for the structure-based design of more potent influenza fusion inhibitors, especially those for H5N1 influenza virus.

## Methods

### Cell Cultures

MDCK cells and 293T cells were obtained from the American Type Culture Collection (ATCC). Cells were grown in Dulbecco’s modified Eagle medium (DMEM, Gibco) containing glutamine, supplemented with 10% fetal calf serum (FCS).

### Site-directed Mutagenesis

A series of single and double mutations were introduced into HA plasmid originated from the A/Qinghai/59/2005 strain by using QuickChange site-directed mutagenesis kit (Stratagene, Cedar Creek, TX), according to the manufacturer’s instructions. The desired mutations were confirmed by nucleotide sequencing of the entire HA coding region.

### Generation of Pseudovirus Bearing Mutant HA and Detection of Pseudovirus Titers

H5N1 pseudoviruses containing mutations M24_1_A, K43_2_A, D46_2_A, G47_2_A V48_2_A, K51_2_A, E105_2_A, R106_2_A, T107_2_A and F110_2_S were generated, as described previously [18]. Briefly, 293T cells (60–70% confluent) were co-transfected with 2 µg of one of the above mutant HA plasmids, 2 µg NA plasmid (pNA), and 3 µg HIV backbone plasmid (pNL4-3.luc.R-E-), which contains an Env and Vpr defective, luciferase-expressing HIV-1 genome, into a six-well plate, using the calcium phosphate precipitation method. Forty-eight hours after the transfection, the culture supernatants were harvested and centrifuged at 2000 rpm for 5 min. Aliquots were stored at −70°C until use. The amount of pseudotyped particles was quantitated using the HIV-1 p24 ELISA kit (Retro-Tek, Buffalo, NY).

### Measurement of the Inhibitory Activity of CL-385319 Against the Infection of Mutant H5N1 Pseudovirus

To measure the inhibitory activity of the test compound against the infection of H5N1 pseudovirus, MDCK cells (1×10^4^/well) were seeded in 96-well plates and grown overnight. CL-385319 at indicated concentration was incubated with pseudotyped particles (1 ng p24/well) for 30 min at 37°C. Subsequently, the virus-compound mixture was transferred to the cells and incubated for an additional 48 h. Cells were washed with PBS and lysed with the lysing reagent included in the luciferase kit (Promega, Madison, WI). Aliquots of cell lysates were transferred to 96-well flat bottom luminometer plates (Costar), followed by addition of luciferase substrate. The luciferase activity was measured in a microplate luminometer (Genios Pro, Tecan, USA).

### Measurement of Hemagglutinin Expression by Western Blotting

Wild-type and mutant hemagglutinin on the surface of the generated pseudoviruses were determined by SDS-PAGE, followed by Western blotting. Briefly, lysed pseudovirus were resolved and run on SDS-PAGE with 10% Tricine gel. The gel was then transferred to nitrocellulose membrane (Roche) and blocked in 5% non-fat milk for 1 h at room temperature. The membrane was incubated with anti-HA mAb (eEnyzme) at 1∶1000 dilution overnight at 4 ^o^C. After three washes, the membrane was incubated with HRP-conjugated goat anti-mouse IgG (1∶2000) for 1 h at room temperature. Signals were visualized with ECL Western blotting substrate reagents.

### Molecular Docking

Mutagenesis data demonstrate that CL-385319 bound to a cavity in the stem region of HA. This cavity is surrounded by the residues G1_2_, L2_2_, K51_2_, E105_2_, R106_2_, T107_2_, L108_2_, D109_2_, F110_2_, T22_1_, M24_1_, E25_1_ and R322_1_, according to the X-ray crystal structure of H5N1 influenza virus HA. The subscripts 1 and 2 refer to HA1 and HA2 subunits, respectively [18]. Based on this information, the AutoDock program was applied to dock CL-385319 into its binding site [38]. The Lamarckian genetic algorithm (LGA) was applied to model the interaction of the molecules with the protein. The docking area was defined using the AutoDock module ADT. The grid site was constrained to a 22.5 Å cubic space centered on the binding site, using a grid point spacing of 0.375 Å. For each molecule, 10 runs were carried out with 150 individuals in the first population and 2.5 million energy evaluations.

### Molecular Dynamics Simulations

The resulting ligand coordinates with HA1 and HA2 subunits were used as starting structures for further energy minimizations, using the Sander module of the Amber11 program, before the final binding structures were achieved. The atomic charges used for CL-385319 were the restrained electrostatic potential (RESP) charges, as determined by using the standard RESP procedure implemented in the antechamber module of the Amber11 program following the electronic structure and electrostatic potential calculations at the HF/6–31G* level [39]. The complex was solvated in a periodic box of TIP3P water molecules that extended 8 Å from the protein and was neutralized by Na^+^ counterions by using the LEaP module, resulting in 20 sodium ions and 27,889 water molecules added [40]. There are a total of 98,819 atoms in the molecular dynamics system. The ff99SB and general AMBER force fields (GAFF) of Amber 11 were used to model the systems.

The SANDER.MPI module in the Amber 11 program was used for the minimization, heating and density protocols. The PMEMD.CUDA module was used for the equilibration and production simulations. Before normal mode calculations, the energy minimization was performed in the gas phase, first for 2000 steps using 2000 steps of the steepest descent (SD) algorithm, and then for another 2000 steps using a conjugate gradient (CG) algorithm. The energy minimized complexes were equilibrated by carrying out 50 ps of heating and 50 ps of density equilibration with weak restraints on the complexes followed by 500 ps of constant pressure equilibration at 300 K and 1 atm pressure. After equilibration, two consecutive production runs were performed with the length of 50 ns each, and coordinates were extracted every 100 ps. Root mean squared deviation (RMSD) analyses were performed using the ptraj module of Amber 11.

All simulations were running with shaking on hydrogen atoms, a 2 fs time step and langevin dynamics for temperature control. Both of the equilibration and production simulations were conducted with NPT ensemble.

### MM_GBSA Calculations

The values of the free energy of binding (Δ*G*_bind_) of each inhibitor were calculated according to the equation:


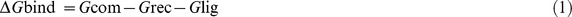


where com, rec and lig stand for complex, receptor and ligand, respectively. The free energy of each of these was estimated as a sum of the four terms:


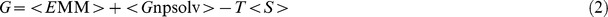


where EMM is the molecular mechanics energy of the molecule expressed as the sum of the internal energy of the molecule plus the electrostatics and van der Waals interactions; Gnpsolv is the polar contribution to the solvation energy of the molecule; Gnpsolv is the nonpolar solvation energy; T is the absolute temperature; and S is the entropy of the molecule.

The nonpolar solvation term (*G*npsolv) was calculated from the solvent accessible surface area (SASA) using the equation:


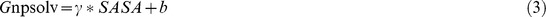


where SASA was determined with the Molsurf method using a probe radius of 1.4Å. Parameter was γ = 0.0072 kcal Å^−2^ and b = 0 kcal/mol to be used with Amber GB polar solvation energies.

Finally, the change in solute entropy during ligand association was estimated by a normal mode analysis of the vibration frequencies and was calculated with the *nmode* module of Amber. Entropies were calculated using the entire protein-ligand complexes. The snapshots for MM_GBSA [41], [42] analyses were taken every 100 ps of the 50 ns to 75 ns MD production runs, resulting in a total of 250 snapshots analyzed. The energies were obtained using the mm_gbsa module of Amber11. The internal, electrostatics and van der Waals energies were calculated with the sander module, with no cutoff for non-bonded interactions. The polar salvation free energies (*G*psolv) were calculated by the generalized Born (GB) approach implemented in Amber11.

### Computational Alanine Scanning (CAS)

CAS replaces a given side chain by an alanine and recalculates the absolute binding free energy for the mutated system. The difference in the binding free energy of the wild-type and alanine mutant, Δ*G*_bind_, may be compared with the results of an experimental AS:


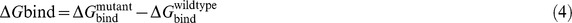


The binding free energy of the alanine mutant is calculated using the MM_GBSA approach, as described earlier, from the set of snapshots obtained for the wild-type complex.

### Charge Density and its Laplacian Calculations

To analyze the bonding characteristics of the complexes, the atoms in molecules (AIM) theory of Bader [43] was applied with the AIM 2000 program package [44]. The AIM theory is based on a topological analysis of the electron charge density and its Laplacian. In the context of quantum calculation of a molecular structure, this theory has proved itself a valuable tool to define the concepts of atom and bonding.

The rigorous AIM theory has been successfully applied in the interpretation of charge density concerning a wide variety of chemical systems [45], [46]. Popelier proposed a set of criteria for the existence of H bonding within the AIM formalism [47], [48]. The most prominent evidence of hydrogen bonding is the existence of a bond path between the donor hydrogen nucleus and the acceptor, as well as a bond critical point (BCP) at which the electron density (**ρ**_b_) ranges from 0.002 to 0.035 au. The AIM calculations in the present study were performed at the B3LYP/6–31+G (d, p) level of theory [39].

## Supporting Information

Movie S1 **The video showing the “induced fit” mechanism of CL-385319 binding to the hemagglutinin.** This video composed hundreds of snapshots through the whole MD simulation, which provides straight-forward information of the distance between F110_2_ and M24_1_ during MD simulation. In the crystal structure of the protein (PDB: 2IBX), the distance between F110_2_ and M24_1_ is 4.235 Å. The first 1200 ps of simulation shown in this video is the MD simulation of hemaggutinin in aqueous solution without CL-385319. At 1200 ps, the distance between F110_2_ and M24_1_ is 5.370 Å, a little larger than that observed from CL-385319-HA complex after energy minimization (5.330 Å), which means the benzene ring of CL-385319 can enter the pocket between F110_2_ and M24_1_. Along with the total 100 ns MD simulation, the distance between F110_2_ and M24_1_ had been increasing, as well as the distance between triﬂuorophenyl group of CL-385319 and V48_2_. Finally, the average distance between F110_2_ and M24_1_ residues increased to 8.950 Å. Other significant conformation changes of residues could not be observed during MD simulation.
(WMV)
